# Segmenting Preventive Health Behavior: Gender Disparities and Psychosocial Predictors in a Culturally Diverse Italian Region

**DOI:** 10.3390/ejihpe15080148

**Published:** 2025-07-31

**Authors:** Dietmar Ausserhofer, Verena Barbieri, Stefano Lombardo, Timon Gärtner, Klaus Eisendle, Giuliano Piccoliori, Adolf Engl, Christian J. Wiedermann

**Affiliations:** 1 Institute of General Practice and Public Health, Claudiana—College of Health Professions, 39100 Bolzano, Italy; 2 Claudiana Research, Claudiana—College of Health Professions, 39100 Bolzano, Italy; 3 Provincial Institute for Statistics of the Autonomous Province of Bolzano—South Tyrol (ASTAT), 39100 Bolzano, Italy; 4 Directorate, Claudiana—College of Health Professions, 39100 Bolzano, Italy

**Keywords:** latent profile analysis, preventive health behavior, patient activation, health literacy, gender differences, mistrust, behavioral segmentation, population-based study, health promotion, social determinants of health

## Abstract

Grounded in health behavior theory, this study examined patterns of preventive health behavior in a culturally diverse, multilingual region of northern Italy using data from a representative population survey (*n* = 2090). Preventive behaviors were assessed using the 16-item Good Health Practices (GHP-16) scale. Latent profile analysis (LPA) identified five behavioral profiles, ranging from ‘Globally Low Engagers’ to ‘Comprehensive High Engagers’. Binary logistic regression compared ‘Globally Low Engagers’ to ‘Broadly Moderate Preventers’, examining predictors including gender, age, education, language, chronic disease status, health literacy (HLS-EU-Q16), patient activation (PAM-10), mistrust of health information, living situation, and healthcare employment. The results showed that men, younger adults, individuals with low patient activation, those living alone, and respondents with high mistrust of health information had higher odds of belonging to the low engagement group. Health literacy and language group membership were not significantly associated with the profile membership. Item-level comparisons revealed gender differences in information-seeking, oral hygiene, and dietary behaviors, with men reporting lower engagement. These findings support a segmentation-based understanding of preventive health behavior and highlight the need to address personal capacities and contextual barriers in interventions while challenging assumptions of uniformly higher female health vigilance.

## 1. Introduction

Preventive health behaviors, including maintaining a healthy diet, engaging in regular physical activity, avoiding tobacco use, and participating in screening programs, are essential for reducing chronic diseases and improving population health outcomes. These behaviors address modifiable risk factors contributing to cardiovascular disease, cancer, diabetes, and other non-communicable conditions that impose substantial burdens on healthcare systems worldwide (Halpin et al., 2010; Corrêa et al., 2024; Gokhale, 2020; Martin Obianyo et al., 2024). Evidence from systematic reviews demonstrates that behavioral interventions, including framing techniques and incentives, can increase adherence to preventive care and improve health outcomes (Corrêa et al., 2024; Martin Obianyo et al., 2024). Additionally, promoting preventive behaviors through public policies and health promotion initiatives has been shown to reduce health disparities among disadvantaged groups (Higgins, 2015; Airhihenbuwa et al., 2021; Da Silva et al., 2024). The promotion of preventive health behaviors remains central to public health strategies for curbing chronic diseases and enhancing health equity.

Health behavior is often explained using theoretical models that describe how individuals make decisions regarding prevention and self-care. Two of the most widely used frameworks are the Health Belief Model (HBM) and Theory of Planned Behavior (TPB).

The HBM focuses on how people weigh the perceived benefits and barriers to taking action, as well as how vulnerable they feel to a given health risk. For example, someone may choose to get a flu shot because they believe it will protect them (benefit), even if they dislike injections (barrier), especially if they feel at high risk due to a chronic condition. Meta-analyses have shown that perceived benefits and barriers are consistently strong predictors of behavior in this model, while perceptions of severity (how serious the illness would be) are less influential (Carpenter, 2010; Sulat et al., 2018).

In contrast, the TPB adds a stronger emphasis on social influences and personal confidence. It explains behavior through three key drivers: behavioral intentions, subjective norms (e.g., what important others think one should do), and perceived control or self-efficacy. For instance, a person may intend to start exercising because they believe it is beneficial, their friends support the idea, and they feel confident that they can do it. The TPB often outperforms the HBM in predicting actual behavior, particularly when intentions and self-efficacy are strong (Gerend & Shepherd, 2012; Montanaro & Bryan, 2014).

Studies that combine elements of the HBM and the TPB often show greater explanatory power than either model alone. This is particularly true when both individual perceptions (such as perceived susceptibility, expected benefits, and perceived control) and social influences (like subjective norms and social support) are taken into account (Huang et al., 2020). While latent profile analysis (LPA) applications are limited here, structural equation modeling using these frameworks identifies subgroups with distinct behavioral profiles, aligning with LPA’s goal of uncovering hidden typologies within populations (Huang et al., 2020). These integrated models provide an improved understanding of behavioral variability and a solid foundation for segmentation approaches targeting interventions.

Health literacy and patient activation are key enablers of preventive health behaviors; however, empirical findings are mixed.

- Higher health literacy is consistently linked to improved preventive practices in chronic disease contexts, including cardiovascular care, COVID-19 adherence, and cancer screening (Gautam et al., 2021; Aaby et al., 2017; Lee et al., 2021). However, its predictive power varies by population and behavior type, with some studies finding no association after controlling for confounders (Mohan et al., 2017).
- Patient activation, which reflects confidence and motivation to manage health, showed weaker associations. While it may enhance health-seeking behavior in some contexts (Erişen, 2024), it is not a reliable predictor of self-care adherence, particularly in chronic disease populations (Meraz et al., 2023).

Studies using behavioral clustering approaches suggest that preventive behaviors polarize into high- and low-adherence groups, and these clusters are better explained by clinical or structural variables than by literacy or activation alone (Zhao et al., 2023). These findings underscore the importance of typology-based analyses that extend beyond linear predictors.

**Figure 1:**
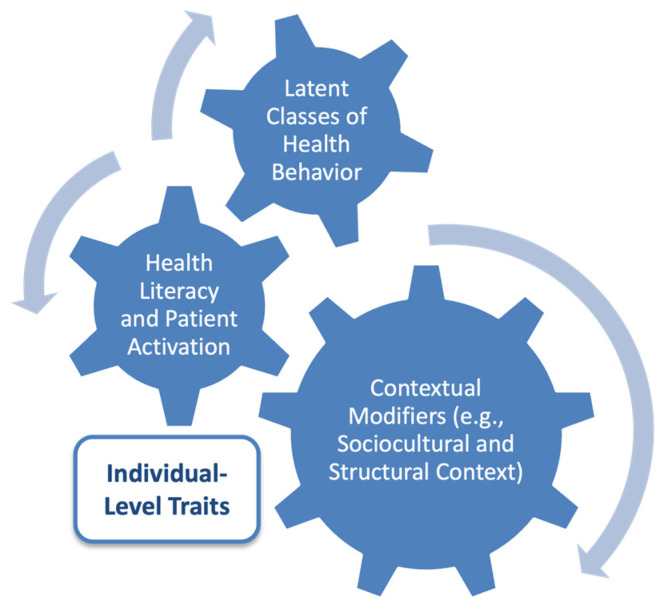
Analytic model guiding the identification of latent profiles of preventive health behavior. This model shows how health literacy and patient activation contribute to distinct latent profiles of preventive health behaviors. These behavioral profiles are shaped by personal capacities and contextual modifiers, including sociocultural and structural factors. The arrows indicate the interactions between the constructs.

Preventive health behaviors are shaped by individual-level factors and broader cultural and linguistic contexts. In multilingual regions of Italy, these factors strongly influence how individuals engage with preventive care and health information. Studies in Northern Italy have highlighted intercultural mediators in bilingual healthcare settings, showing that cultural assumptions shape health-related communication and behavior (Anderson & Cirillo, 2020). A multicenter trial demonstrated that co-designed, culturally tailored interventions improved lifestyle behaviors and therapy adherence among immigrant patients with type 2 diabetes (Bonvicini et al., 2025). Digital and community-based health promotion initiatives targeting disadvantaged youth have shown promise in improving health literacy and preventive knowledge (Franceschi et al., 2021). In South Tyrol, linguistic group membership has been linked to patterns of health information use, with German speakers favoring traditional medical sources and Italian speakers engaging more with digital platforms (Ausserhofer et al., 2022; Wiedermann et al., 2025). These findings support the relevance of culturally sensitive, segmentation-based approaches to behavioral health research. While the importance of preventive health behaviors has been established, current research often relies on linear models that overlook the complexity of behavioral patterns across populations. In culturally diverse contexts like South Tyrol, segmentation approaches like LPA can uncover distinct behavioral profiles hidden by aggregate methods (Figure 1). Research suggests that health literacy and patient activation may not uniformly predict preventive engagement, and that sociocultural context shapes how individuals access, interpret, and act on health information (Smith et al., 2013; Couture et al., 2018; Palumbo, 2021).

Guided by the HBM, which emphasizes perceptions of susceptibility, benefits, and barriers, this study applied LPA to identify subgroups within a representative adult population based on preventive behaviors. The aim was to characterize these behavioral profiles and explore their associations with sociodemographic factors, health literacy, and patient activation, laying the groundwork for tailored interventions in multilingual and culturally heterogeneous settings.

## 2. Methods

### 2.1. Study Design and Sampling

This analysis was part of a population-based cross-sectional survey conducted in the Autonomous Province of Bolzano (South Tyrol), Italy, between March and May 2024. The province is characterized by cultural and linguistic heterogeneity, with German, Italian, and Ladin-speaking communities and a decentralized health system. The Provincial Institute of Statistics (ASTAT) designed the survey in collaboration with the Institute of General Practice and Public Health. Earlier studies using this dataset investigated the associations between mistrust and sleep quality (Wiedermann et al., 2025) and health information behavior (Ausserhofer et al., 2025). The current study focuses on behavioral typologies related to preventive health behaviors.

A total of 4000 individuals (≥18 years) were randomly selected from the provincial population registry to provide robust estimates across strata and enable post-stratification weighting using ReGenesees software (version 2.3, ISTAT, Rome, Italy). The stratification criteria included age group (18–34, 35–54, and 55+ years), sex, citizenship, and municipality of residence. Participants received paper-based or online survey invitations in German and Italian. Of the 4000 individuals invited, 2120 responded, yielding a response rate of 53%. After data cleaning, 2090 cases were included in the analysis, corresponding to a completion rate of 98.6%. All respondents provided informed consent, and participation was voluntary and anonymous. The present analysis was based on 2090 completed responses.

### 2.2. Sociodemographic and Health Measures

Participants reported their biological sex, age (in years), and language group, with response options for German, Italian, Ladin, or others. For analytical purposes, the Ladin and “Other” groups were combined. Educational attainment was recorded in four categories: primary school, vocational/technical education, high school diploma, and university degrees.

Living situation was captured using a binary item indicating whether the respondent lived alone or with others (e.g., family or partner). Chronic illness status was assessed using a self-reported checklist. Respondents were asked whether they had ever been diagnosed with a range of common chronic conditions (e.g., cardiovascular disease, hypertension, diabetes, and mental health disorders). For the present analysis, a dichotomous variable (presence or absence of any condition) was constructed. Additional variables included employment in the health or social care sector (yes/no). For operational definitions of these and other background variables, readers are referred to prior methodological reports from this study series (Ausserhofer et al., 2025; Wiedermann et al., 2025).

### 2.3. Preventive Health Behavior (GHP-16)

Preventive behavior was assessed using the Good Health Practices scale (GHP-16) (Hampson et al., 2019), a validated short-form inventory derived from the original Health Behavior Checklist (HBC) (Vickers et al., 1990). The GHP-16 measures engagement in 16 diverse health-promoting practices across the lifestyle, medical, and hygiene domains. Items include:

- “I exercise regularly”,
- “I follow a balanced diet”,
- “I get routine medical check-ups”,
- “I floss my teeth regularly”, and
- “I avoid smoking” (phrased as “I don’t smoke”).

Each behavior was rated on a 4-point Likert scale ranging from 1 (“does not apply at all”) to 4 (“fully applies”). To facilitate comparison with international studies using a 5-point format, responses were linearly rescaled using the following formula: rescaled score = 1 + (original score − 1) × (4/3). This preserved the ordinal structure while aligning the scale values with previously published work on the GHP-16. Higher scores indicate more consistent engagement in the preventive practices. No reverse coding was required because all items were positively phrased. The total score was computed as the arithmetic mean of non-missing items, resulting in a scale ranging from 1.0 (minimal engagement) to 5.0 (maximum engagement). The original GHP-16 validation study reported an internal consistency of α = 0.83 (Hampson et al., 2019). In our analytic sample, the internal consistency of the GHP-16 was acceptable (Cronbach’s α = 0.77). Participants missing more than two items were excluded from the score-based analyses.

The GHP-16 items were translated into German and Italian by the ASTAT team using standard translation procedures, including pretesting and back-translation. However, a formal psychometric validation of the translated versions was not conducted. Therefore, observed differences between language groups may, in part, reflect language- or culture-specific interpretation effects. The GHP-16 served two purposes in this study: (1) as the input for latent profile analysis, modeling patterns of behavior across the 16 items, and (2) as the basis for item-level gender comparisons in descriptive analyses.

### 2.4. Health Literacy (HLS-EU-Q16)

Health literacy was measured using the 16-item European Health Literacy Survey Questionnaire (HLS-EU-Q16). The instrument captures the perceived difficulty in accessing, understanding, appraising, and applying health-related information. Items include:

- “How easy is it for you to understand what your doctor says to you?”
- “How easy is it for you to judge which everyday behaviors are related to your health?”
- “How easy is it for you to find information about treatments of illnesses that concern you?”

Validated Italian and German versions of the HLS-EU-Q16 were used in this study, as developed and published by (Lorini et al., 2019) and (Pelikan et al., 2014), respectively. Each question was rated on a four-point Likert scale (1 = very difficult to 4 = very easy). In accordance with the scoring guidelines (Lorini et al., 2019; Pelikan et al., 2019; Sørensen et al., 2015), each response of “very easy” or “easy” was scored as 1, while “difficult” and “very difficult” were scored as 0. The total score was computed as the sum of the valid items, resulting in a summary index ranging from 0 (low health literacy) to 16 (high health literacy). Respondents were required to answer at least 14 items to obtain valid scores. Those with fewer valid responses were categorized as “missing” for health literacy and retained in all models as separate categories. Based on standard cutoffs, valid scores were categorized as follows: 0–8, inadequate; 9–12, problematic; and 13–16, sufficient. These categories were used for all group-wise and regression analyses. The internal consistency of the HLS-EU-Q16 has been previously reported as α = 0.82 for the German version (Pelikan et al., 2014) and α = 0.84 for the Italian version (Lorini et al., 2019). In our analytic sample, Cronbach’s alpha for the HLS-EU-Q16 was 0.89, indicating high internal consistency.

### 2.5. Patient Activation (PAM-10)

Patient activation was assessed using the PAM-10, a validated 10-item short form of the Patient Activation Measure. The instrument captures an individual’s confidence, motivation, and self-management skills in relation to healthcare and daily routines. Sample items include:

- “I know how to prevent further problems with my health”
- “I can stick to positive health routines even during stress”
- “I am confident that I can tell my doctor concerns I have even when he or she does not ask.”

Items were answered on a 4-point Likert scale, with higher scores indicating greater agreement. The raw scores were converted to a 0–100 scale using the standard conversion table provided by Insignia Health (Brenk-Franz et al., 2013; Graffigna et al., 2017; Lin et al., 2023). We used the validated German (PAM13-D) and Italian (PAM13-I) versions of the instrument, which have demonstrated robust psychometric properties in previous studies and were adapted for 10-item scoring. Participants were then categorized into four levels of activation: Level, disengaged and overwhelmed; Level 2, becoming aware but struggling; Level 3, taking action; and Level 4, maintaining behaviors and pushing further. These ordinal categories were used to examine the differences in health behavior profiles and predictors of profile membership. Cronbach’s alpha for the PAM–10 was 0.86 (Lin et al., 2023). In our sample, Cronbach’s alpha for the PAM-10 was 0.81, supporting its reliability.

### 2.6. Mistrust of Professional Health Information

Perceived mistrust in formal health information was assessed using a previously described 4-item Mistrust Index (Wiedermann et al., 2025). Participants rated their trust in general practitioners, outpatient specialists, pharmacists, and nurses on a 4-point Likert scale ranging from 1 (“very trustworthy”) to 4 (“not at all trustworthy”). Item scores were summed to yield a composite index ranging from 4 to 16, with higher values indicating greater mistrust of professional health sources.

### 2.7. Statistical Analysis

Statistical analyses were conducted using a combination of IBM SPSS Statistics Version 29.0.2.0 (20) (IBM Corp., Armonk, NY, USA) and RStudio Version 2025.05.1+513 (Posit, PBC, Boston, MA, USA). General descriptive, comparative, and regression analyses were implemented in SPSS, while latent profile analysis was performed in R.

#### 2.7.1. Latent Profile Analysis

Latent profile analysis (LPA) was used to identify behavioral subgroups based on the 16 rescaled items from the GHP-16. The items were treated as continuous indicators. Models with 1 to 6 latent profiles were estimated using Gaussian finite mixture models assuming profile-invariant variances and zero covariances, implemented via the R package (tidyLPA v1.1.0) (R Foundation for Statistical Computing, Vienna, Austria). Model estimation relied on the Expectation-Maximization (EM) algorithm with default convergence settings.

Model selection followed current best practice guidelines (Van Lissa et al., 2023), considering Bayesian Information Criterion (BIC), Integrated Completed Likelihood (ICL), and classification precision. The final model was selected based on a balance of statistical fit and substantive interpretability. To assess measurement quality, average posterior probabilities (AvePP) for profile membership were examined, with values ≥ 0.70 indicating adequate assignment quality. The tidyLPA package used for latent profile analysis, based on the mclust framework, does not provide LMR or BLRT tests for determining the number of classes. Consequently, model selection was based on BIC, ICL, and AvePP, as recommended (Van Lissa et al., 2023).

#### 2.7.2. Descriptive and Comparative Statistics

Descriptive statistics were computed for all the variables included in the analysis. Means and standard deviations (SD) were reported for continuous variables, such as GHP-16 item scores, while proportions and absolute counts were reported for categorical variables.

To compare behavioral patterns across groups, gender-stratified analyses of GHP-16 item responses were conducted using Mann–Whitney U tests, as the item-level data showed non-normal distribution. The corresponding effect sizes were calculated using Cliff’s delta.

To assess the potential impact of common method variance (CMV) due to the use of self-reported measures in a cross-sectional design, we conducted Harman’s single-factor test. An unrotated exploratory factor analysis was performed, including all items from the GHP-16, PAM-10, and HLS-EU-Q16 instruments. The first factor accounted for 16.13% of the total variance, and ten factors had eigenvalues greater than 1. These results suggest that CMV is not a serious concern in this dataset.

#### 2.7.3. Binary Logistic Regression Analysis

To examine predictors of minimal engagement in preventive health behaviors, a binary logistic regression analysis was conducted comparing individuals assigned to Profile 5 (‘Globally Low Engagers’) versus Profile 2 (‘Broadly Moderate Preventers’) of the five-profile latent profile solution. This contrast was chosen due to its public health relevance: Profile 5 members reported the lowest overall engagement across the GHP-16 behavioral domains, whereas Profile 2 represented a moderately engaged and demographically broad reference group.

The binary outcome variable was coded as 1 for ‘Globally Low Engagers’ and 0 for ‘Broadly Moderate Preventers’. All other cases were excluded from the analysis.

The following predictors were included based on theoretical and empirical relevance: Gender, age, education level, language group, living arrangement (living alone vs. with others), presence of chronic disease, health literacy score, patient activation score, and mistrust of health information. All categorical variables were dummy coded using the lowest engagement or most neutral category as the reference level. Continuous variables were treated as linear predictors unless assumptions were violated. The final model included only participants with complete data on all covariates.

The regression model was estimated using the Binary Logistic Regression procedure in SPSS with the enter method. Results are reported as odds ratios (Exp(B)) with 95% confidence intervals and *p*-values. Model fit was assessed using the Hosmer–Lemeshow goodness-of-fit test. To ensure the validity of model assumptions, a series of diagnostic checks were conducted: Linearity of the logit was tested for continuous predictors (age, health literacy, patient activation, mistrust) using the Box–Tidwell transformation. Non-significant interaction terms (*p* > 0.05) confirmed the assumption of linearity. Independence of residuals was approximated using a Durbin–Watson statistic from a linear regression with the same covariates. The value was within the acceptable range (1.5–2.5). Multicollinearity was evaluated through Variance Inflation Factors (VIFs), which were all below 2.5. Outlier and influence diagnostics were performed using standardized residuals (flagged if >|3.0|) and Cook’s distance (flagged if >1.0). No influential cases were found that warranted exclusion.

## 3. Results

### 3.1. Latent Profile Analysis of Preventive Health Behavior

To identify subgroups based on patterns of preventive health behavior, LPA was conducted using the 16 rescaled items of the GHP-16 as continuous indicators. Models with one to six latent profiles were estimated using

Model selection was guided by the BIC, interpretability, and parsimony. Although the 6-profile solution yielded the lowest BIC (−97,250), the 5-profile solution (BIC = −97,344) was selected as the optimal model due to its clearer conceptual distinction between profiles and better distribution of participants. All profiles contained more than 10% of the sample, and entropy-based classification quality was high, with average posterior AvePP ranging from 0.835 to 1.000, indicating strong assignment certainty. Although the 6-profile model had a marginally lower BIC, its substantive interpretability and profile distinctiveness were inferior to the 5-profile solution.

The five behavioral profiles were labeled as follows:

- Profile 1—‘Medically Compliant, Lifestyle Passive’ (29.2%): The largest profile, characterized by high scores for vaccinations and medical check-ups but lower engagement in exercise, supplementation, and information-seeking.
- Profile 2—‘Broadly Moderate Preventers’ (22.9%): Showed moderate engagement across most domains, with strengths in oral hygiene and sleep.
- Profile 3—‘Comprehensive High Engagers’ (20.6%): Exhibited uniformly high scores across lifestyle and medical prevention, reflecting a broadly health-conscious profile.
- Profile 4—‘Peripherally Engaged’ (16.7%): Displayed modest engagement across domains, with particularly low use of preventive dental and screening services.
- Profile 5—‘Globally Low Engagers’ (10.7%): Demonstrated the lowest scores on nearly all behaviors, including diet, supplementation, and preventive visits.

Profile assignment was based on the highest posterior probability for each individual. Table 1 presents the item-level means and SDs for each profile.

### 3.2. Sociodemographic and Health Characteristics by Behavioral Profile

Using population-weighted data, Table 2 presents the distribution of key sociodemographic and health-related characteristics across the five latent profiles of preventive health behavior. Several characteristics varied significantly across profiles, but only a few reached effect sizes indicative of practical relevance.

Gender emerged as the strongest profile-defining characteristic, with a moderate effect size (Cramér’s V = 0.241). Age group and patient activation were also associated with profile membership, each with small-to-moderate effect sizes (Cramér’s V = 0.144 and 0.127, respectively). In contrast, differences in language group, educational attainment, chronic disease status, and health literacy level, while statistically significant, showed only small or negligible effect sizes, suggesting limited contribution to profile differentiation.

#### 3.2.1. Post Hoc Comparisons

Post hoc pairwise comparisons using adjusted standardized residuals (Bonferroni-corrected threshold: |z| > 2.81) revealed significant overrepresentation of men in the ‘Broadly Moderate Preventers’ and ‘Globally Low Engagers’ profiles, and of women in the ‘Comprehensive High Engagers’ profile. These contrasts confirm gender as a central driver of behavioral segmentation.

Age group differences further supported profile distinctions: the ‘Medically Compliant, Lifestyle Passive’ profile was dominated by older individuals (55+), while the ‘Peripherally Engaged’ and ‘Broadly Moderate Preventers’ profiles included disproportionately more young adults (18–34). These patterns suggest age-related gradients in engagement, particularly with younger individuals clustering in less medically anchored profiles.

With respect to patient activation, the ‘Comprehensive High Engagers’ had significantly more individuals at the highest PAM level (z = 7.5), whereas both the Broadly Moderate and ‘Globally Low Engagers’ were more likely to be found in the lowest activation category. This highlights a meaningful relationship between behavioral engagement and self-management capacity, albeit with a small overall effect size.

#### 3.2.2. Health Literacy

Differences in health literacy scores across behavioral profiles were assessed using both the non-parametric Kruskal–Wallis test and one-way ANOVA, given the non-normality of the data. Given the robustness of ANOVA to moderate deviations from normality in large samples, and the similar results across both approaches (Table 2), post hoc comparisons were based on Bonferroni-adjusted ANOVA tests.

A one-way ANOVA revealed a significant effect of behavioral profile on health literacy scores, F(4, 1644) = 11.06, *p* < 0.001, with a small effect size (η^2^ = 0.026). Bonferroni-corrected post hoc tests indicated that the ‘Medically Compliant, Lifestyle Passive’ group had significantly higher HLS scores than the Broadly Moderate, ‘Comprehensive High Engagers’, and Globally Low Engagers. The ‘Peripherally Engaged’ profile also scored significantly higher than both the Broadly Moderate and Globally Low groups. These findings suggest that while health literacy varies significantly across behavioral profiles, it only modestly contributes to distinguishing low engagement from medically oriented subgroups.

### 3.3. Gender Differences in Individual Preventive Health Behaviors

Table 3 presents a population-weighted comparison of preventive health behavior items between women and men. Although several behaviors showed statistically significant differences, most of the observed effects were small or negligible in practical terms.

The strongest gender differences, indicated by small-to-medium Cliff’s delta values, were observed in behaviors associated with information engagement, supplementation, and communication. Men reported higher frequencies of gathering health information before making decisions, talking with others about health, and taking vitamins or supplements to prevent illness. These differences, although not large, suggest that male participants may engage more actively in informational and instrumental self-care strategies than commonly assumed.

Differences were also evident in the dietary and oral health practices of the participants. Men reported higher engagement in following a balanced diet, limiting the intake of high-fat or sugary foods, and visiting the dentist regularly. They also showed modestly higher adherence to routine checkups and oral hygiene behaviors, such as flossing and brushing. These patterns contribute to the broader preventive engagement profiles previously observed in profil-based analyses and challenge gender stereotypes that typically associate women with greater health vigilance than men.

In contrast, smoking abstinence, physical activity, and sleep quality showed minimal or negligible gender differences. Self-reported exercise and sleep patterns were nearly identical between the sexes, and the already high levels of non-smoking behavior in both groups showed only minor variation.

Taken together, these findings confirm that while men and women differ in specific preventive behaviors, particularly in health information use and dietary vigilance, the overall magnitude of gender differences at the item level was limited.

### 3.4. Predictors of Low Preventive Engagement: Profile 2 (‘Broadly Moderate Preventers’) vs. Profile 1 (Globally Low Engagers)

To identify characteristics associated with minimal engagement in preventive health behaviors, a binary logistic regression was conducted comparing Profile 5 (‘Globally Low Engagers’) with Profile 2 (‘Broadly Moderate Preventers’) based on the five-profile latent profile solution (Table 4). A total of *n* = 577 participants with complete data on all predictors were included (‘Globally Low Engagers’: *n* = 266, BMP: *n* = 311).

The model was statistically significant (χ^2^(12) = 35.90, *p* < 0.001) and explained 8.1% of the variance in profile membership (Nagelkerke R^2^ = 0.081). The Hosmer–Lemeshow test indicated acceptable model fit (χ^2^(8) = 12.25, *p* = 0.140), and the Durbin–Watson statistic from an auxiliary linear regression was 1.90—suggesting independence of residuals.

Linearity of the logit was tested using the Box–Tidwell procedure; none of the interaction terms with log-transformed predictors were statistically significant, confirming the assumption of linearity. No standardized residuals exceeded |3.0|, and the maximum Cook’s Distance was well below 1 (max = 0.12), indicating the absence of influential outliers.

Age was a significant predictor: with each additional year, the odds of being in the ‘Globally Low Engagers’ group decreased by 1.7% (OR = 0.983, *p* = 0.004). Living alone was associated with higher odds of ‘Globally Low Engagers’ membership (OR = 1.80, *p* = 0.011). Chronic disease was also predictive: individuals with chronic conditions had increased odds of being disengaged (OR = 1.79, *p* = 0.004).

Gender, language group, and patient activation showed trends but did not reach statistical significance in the multivariable model. Health literacy and mistrust were not significantly associated with profile membership.

These findings suggest that structural vulnerabilities, particularly living alone, chronic illness, and younger age, are stronger predictors of globally low preventive engagement than motivational constructs such as activation or trust.

## 4. Discussion

This study identified five distinct latent profiles of preventive health behavior using 16 items from the GHP-16 scale in a representative sample of adults in South Tyrol. The profiles ranged from globally low engagement (Profile 5) to high engagement across all domains (Profile 3), with the largest share of participants falling into the medically compliant but lifestyle-passive group (Profile 1). Patient activation showed a clear gradient across profiles, whereas health literacy and language groups had limited explanatory power. Binary logistic regression confirmed that younger age, living alone, and chronic disease were significant predictors of globally low engagement. Among these, younger age showed the strongest associations. These findings underscore the need to examine not only direct predictors but also potential interactions between personal capacities and contextual barriers in preventive health research.

### 4.1. Interpretation Within a Health Behavior Framework

The five behavioral profiles align with the study’s conceptual model (Figure 1). In this model, individual factors like motivation, confidence, and self-management interact with social and structural conditions to shape different patterns of preventive behavior (Hagger, 2025). Instead of viewing preventive behavior as a direct result of knowledge or demographics, the profile structure reveals meaningful subgroups with distinct patterns of behavior (Hill et al., 2022; L. K. Tong et al., 2024).

Beyond statistical fit indices, the five-profile solution was also supported by its substantive interpretability and external validity. Each profile exhibited a distinct and theoretically meaningful pattern of behavior, such as Profile 2’s across-the-board disengagement or Profile 3’s passivity in self-care but not in medical checkups. Additionally, the profiles correlated in expected directions with age, gender, mistrust, and patient activation, factors well-established in health behavior research. These patterns, and their practical value for designing public health interventions, support the external validity of the model.

The contrast between Profile 2 (Globally Low Engagers) and Profile 1 (‘Broadly Moderate Preventers’) offers insights into the determinants of poor preventive engagement. Individuals in Profile 2 were significantly more likely to be male, younger, less activated, and to report higher levels of mistrust in professional health information. In contrast, those in Profile 1 tended to be older, more often female, and reported higher patient activation. These patterns align with the analytic model’s dual emphasis on personal capacities (such as activation and self-regulation) and contextual modifiers, including mistrust and social isolation (Hagger, 2025; L. K. Tong et al., 2024). Those living alone had significantly higher odds of belonging to Profile 2, although the effect size was moderate

Gender was the strongest predictor of profile membership. Men were overrepresented in the least engaged behavioral profile and underrepresented in the moderate- and high-engagement profiles. This finding challenges the common belief that women are always more health-conscious than men. Although health literacy is often conceptualized as a key predictor of health behavior, it was not significantly associated with profile membership in this study. This disconnect supports the notion that knowledge alone is insufficient to ensure safety. Motivation, trust, and social context play equally critical roles in shaping actions (Young-Silva et al., 2024; Bermeo et al., 2023).

The item-level analyses of GHP-16 behaviors support this interpretation in the following ways. Men reported lower engagement in health information-seeking, oral hygiene, and dietary control behaviors, which were aligned with the moderate- and high-engagement profiles. However, they reported higher frequencies of behaviors such as dietary supplement use or discussing health. These findings show the value of behavior-based segmentation and question common gender stereotypes in preventive health. Rather than assuming uniform needs across demographic groups, these results support the development of tailored interventions that align with empirically derived behavioral profiles (Burgermaster & Rodriguez, 2022).

Cultural-linguistic identity, captured by the language group, showed minimal association with the behavioral profiles. While language may influence communication preferences or trust in health information—as shown in previous analyses—it appears less relevant in shaping the structure of preventive behavior. This reinforces the idea that sociocultural variables act more as moderators of access and perception than as direct determinants of behavioral engagement (Short & Mollborn, 2015).

The observation that men reported both lower overall engagement and higher frequencies of specific behaviors like supplement use and health discussions warrants examination. Gendered reporting tendencies or social desirability bias may contribute to these patterns (Oksuzyan et al., 2019). Men might overreport proactive behaviors when asked directly, while underreporting routine activities like oral hygiene. Women, however, might provide more accurate self-assessments across behaviors. These response biases highlight the need to triangulate self-report data with objective measures and avoid overgeneralizing sex-based patterns without context.

### 4.2. Socio-Behavioral Drivers of Low Engagement

The regression analysis comparing Profile 2 (Globally Low Engagers) to Profile 1 (‘Broadly Moderate Preventers’) identified individual and psychosocial characteristics linked to low preventive health behaviors. Although several sociodemographic variables were statistically significant, few were meaningful predictors of disengagement. Gender differences were notable: men had higher odds of being ‘Globally Low Engagers’ than women, indicating that male participants were overrepresented in the least engaged profile (Laska et al., 2009; Tabacchi et al., 2018). Age also influenced profile membership, with older individuals (especially those aged 55 and above) less likely to be in Profile 2, suggesting that preventive disengagement is more common among younger men, aligning with research on life-stage health behavior influences (Collins et al., 2023).

Among psychosocial variables, mistrust of professional health information was the main driver of disengagement. Individuals with higher Mistrust Index scores had increased odds of being Globally Low Engagers, reinforcing that low trust in health professionals can undermine engagement with recommended practices (Jaiswal & Halkitis, 2019; White et al., 2016). This finding suggests that rebuilding trust, especially among younger men, may be essential for improving adherence. Social isolation, as indicated by living alone, was linked to low engagement. Those living alone were nearly three times more likely to be in Profile 2 than those living with others. This supports including social context in behavior models and shows how living alone can affect habits like diet, hygiene, or medical visits (Dreyer et al., 2018; Hanna & Collins, 2015).

Patient activation findings confirm the role of motivation and self-efficacy in adopting preventive behaviors (Smith et al., 2013; Hibbard, 2017). In contrast, health literacy, both as a categorical and continuous measure, did not meaningfully predict profile membership. Sensitivity analysis confirmed only negligible differences in HLS scores across profiles, further suggesting that informational competence alone does not translate into behavioral engagement when trust or personal agency are lacking (Meraz et al., 2023). This shows that knowing what to do is insufficient without perceived control, motivation, and supportive environments.

These findings indicate that low preventive engagement reflects an interaction between distrust, isolation, and low agency, beyond sociodemographic disadvantages. Interventions addressing these barriers may be more effective than generic educational campaigns. Engaging community peer supporters or targeting health messages through trusted intermediaries could prove more successful in shifting behavior among low-engagement groups (Schwartz & Sendor, 1999).

### 4.3. Practical and Regional Implications

One unexpected finding was the minimal role of the language group in shaping preventive health behavior profiles. Despite South Tyrol’s linguistic diversity, where German, Italian, and Ladin speakers coexist, language group membership did not meaningfully predict profile membership in the latent profile model or regression analysis. This contrasts with earlier analyses showing differences in health information-seeking by language group (Ausserhofer et al., 2022). The findings suggest that while cultural identity may influence how individuals access health information (Zanchetta & Poureslami, 2006), it plays a lesser role in preventive behaviors when controlling for individual and psychosocial factors (Solis et al., 1990; Unger, 2011).

This has implications for the generalizability of the findings. Although situated in a unique region, the identified behavioral profiles appear to transcend local language boundaries. This segmentation approach could also be useful in other multilingual regions, like Switzerland. This finding reinforces that preventive behaviors are shaped more by psychological and structural factors such as mistrust, isolation, and perceived self-efficacy than by cultural affiliation (Yap et al., 2023).

Latent profile segmentation helps public health planning by identifying behavioral subgroups that need tailored interventions. ‘Globally Low Engagers’ may need trust-building and social support (Drageset, 2021; Wilkins, 2018), while High Engagers may benefit from feedback and reinforcement (Krukowski et al., 2020; DiClemente et al., 2001). Medically Passive individuals may require lifestyle coaching despite adequate preventive check-up behavior (Conn & Curtain, 2019).

This approach challenges the assumptions underlying health communication strategies. The finding that health literacy did not distinguish behavioral profiles, while mistrust and activation levels did, shows the need for context-sensitive messaging targeting psychological readiness (Smith et al., 2013). This may include peer-based outreach or programs that build health-related confidence rather than just transmitting knowledge (Brinsley et al., 2025).

Segmenting the population into behavioral subgroups can enhance equity in health promotion. By identifying groups with the greatest need, such as younger men with high mistrust and limited social connections, interventions can be better targeted, ensuring that resources are used efficiently and fairly to maximize their public health impact.

The identified profiles suggest distinct intervention needs. ‘Globally Low Engagers’ may require trust-building and social support through outreach by community intermediaries and messaging focused on small, achievable steps (Ward, 2017). Medically Passive individuals might benefit from personalized feedback and low-threshold digital tools to support lifestyle changes beyond routine check-ups (H. L. Tong et al., 2021). Aligning strategies with behavioral profiles may enhance the relevance and impact of preventive efforts.

### 4.4. Strengths and Limitations

This study had several methodological strengths that enhanced its validity. First, it used a large, population-based stratified sample of South Tyrolean adults, with a standardized bilingual survey design supporting representativeness. The stratification across sociodemographic variables and calibrated weights ensured that the results reflected the general adult population. Second, the LPA of the GHP-16 items identified empirically derived behavioral profiles beyond traditional scoring methods. Third, the study included comprehensive psychosocial and contextual variables, including mistrust, living situation, and patient activation, enabling an integrative analysis of behavioral engagement reflecting individual and social health determinants.

However, there are some limitations to this approach. The analysis relied on self-reported behaviors that were subject to recall bias and social desirability effects. While the GHP-16 shows validity, self-assessments may not align with objective behaviors. The use of rescaled Likert data for GMM could limit comparability with the categorical LPA techniques.

LPA and binary logistic regression used unweighted data. Although this enhances the LPA model stability, it limits the generalizability of profile proportions. Population weights were applied post hoc to improve the representativeness of descriptive profiling and comparisons. Additionally, the software framework used for LPA estimation (tidyLPA, based on mclust) does not provide Lo-Mendell-Rubin (LMR) or bootstrap likelihood ratio tests (BLRT) for determining the number of profiles. Therefore, model selection relied on BIC, Integrated Completed Likelihood, and AvPP, in line with current recommendations. Furthermore, we did not apply BCH-adjusted comparisons when analyzing differences across profiles, which would account for classification uncertainty. While our approach was based on direct class assignment using maximum posterior probabilities, future studies could enhance precision by implementing BCH methods now available in packages like tidySEM.

Furthermore, the regression model did not include certain key structural determinants such as household income, employment type, or healthcare access, as these were not assessed in the survey. The absence of these variables may limit the explanatory power of the predictive model and hinder deeper understanding of the economic or occupational drivers of low preventive engagement. Future research should aim to integrate these dimensions to better capture the full range of social determinants influencing preventive health behavior.

The cross-sectional design precludes causal inferences about behavioral changes. The relationship between mistrust, activation, and preventive behaviors is still hypothetical. Additionally, factors such as health service access and income were not fully explored, although they may affect behavioral segmentation.

Despite these limitations, this study provides an empirical framework for understanding preventive engagement patterns and supports segmentation models in research and public health planning.

## 5. Conclusions

This study identified five distinct behavioral profiles of preventive health engagement in a population-based sample from South Tyrol using latent profile analysis of the GHP-16. While most individuals exhibited moderate or high levels of engagement, a small but important subgroup—Globally Low Engagers—emerged with consistently low scores across preventive domains. This group was disproportionately composed of younger, less activated men and individuals with higher mistrust of health professionals or limited social ties.

The results highlight that preventive actions are influenced not only by personal knowledge or demographic factors but also by a combination of individual abilities (such as confidence and self-management) and contextual factors (such as social isolation and trust in personal health information). The lack of a strong link between health literacy and behavioral categories indicates that information alone is insufficient to effect behavior change. Motivation, perceived control, and trust are the essential drivers.

This study lays the groundwork for more focused and fair health promotion strategies by dividing the population into groups with distinct behaviors. Efforts to enhance preventive actions should consider not only people’s knowledge, but also their beliefs, the level of control they perceive they have, and the environment they inhabit. Employing person-centered, data-driven segmentation models, such as LPA, could pave the way for crafting more adaptive and successful prevention initiatives in South Tyrol and other similar contexts.

## Figures and Tables

**Table 1:** GHP-16 scores per latent profile of preventive health behavior (*n* = 2090 participants).

Health Behavior (GHP-16 Item)	GHP-16 Scores, Mean (SD)				
	Medically Compliant, Lifestyle Passive	Broadly Moderate Preventers	Comprehensive High Engagers	Peripherally Engaged	Globally Low Engagers
*n*	610	478	431	348	223
1—Exercise regularly	2.82 (1.16)	2.93 (1.18)	3.67 (1.11)	2.57 (1.05)	3.64 (1.05)
2—Follow a balanced diet	3.35 (1.14)	3.35 (1.06)	4.16 (0.86)	3.04 (1.02)	4.12 (0.78)
3—Take vitamins or dietary supplements	2.26 (1.17)	2.00 (1.06)	3.89 (0.94)	2.09 (1.05)	2.14 (1.00)
4—Visit the dentist regularly	3.35 (1.36)	3.05 (1.29)	4.13 (1.12)	2.78 (1.16)	4.20 (1.02)
5—Watch weight or try to lose weight	2.92 (1.18)	2.58 (1.07)	3.73 (1.10)	2.70 (1.03)	3.56 (1.06)
6—Limit intake of high-fat or high-sugar foods	2.84 (1.06)	2.86 (0.97)	3.76 (0.91)	2.79 (1.00)	3.61 (0.86)
7—Gather health information before making decisions	2.92 (1.14)	2.45 (0.97)	3.91 (0.91)	2.66 (1.01)	3.39 (1.00)
8—Pay attention to physical symptoms or health changes	3.45 (1.12)	3.07 (1.05)	4.26 (0.87)	3.31 (0.97)	4.27 (0.82)
9—Take supplements to prevent illness	1.92 (1.17)	1.55 (0.85)	3.86 (0.89)	1.81 (0.97)	1.62 (0.72)
10—Get routine medical check-ups	2.78 (1.11)	2.45 (0.93)	3.60 (1.17)	2.72 (1.03)	3.50 (1.10)
11—Floss teeth regularly	2.59 (1.43)	2.07 (1.21)	3.32 (1.44)	1.86 (0.98)	3.13 (1.45)
12—Talk with others about health	2.93 (1.06)	2.57 (0.93)	3.56 (0.92)	2.75 (1.03)	3.27 (0.93)
13—Don’t smoke	1.54 (0.66)	4.86 (0.41)	4.91 (0.33)	4.87 (0.39)	4.91 (0.33)
14—Brush teeth twice a day	4.64 (0.80)	5.00 (0.00)	4.91 (0.33)	3.36 (0.67)	4.93 (0.30)
15—Get vaccinated	3.40 (1.31)	3.19 (1.29)	3.62 (1.29)	3.29 (1.18)	3.83 (1.23)
16—Get enough sleep	3.58 (1.01)	3.64 (0.93)	3.88 (0.92)	3.58 (0.94)	3.95 (0.89)

Values represent means and SDs (in parentheses) for each of the 16 preventive health behavior items from the Good Health Practices scale (GHP-16), stratified by latent profile membership. Higher scores indicate more frequent or consistent engagement in the behavior. Profile membership was derived from a latent profile analysis using Gaussian mixture modeling. The profile labels are arbitrary and do not imply ordering.

**Table 2:** Population-based sociodemographic and health characteristics across latent profiles of preventive health behavior (*n* = 2090).

Variable	Medically Compliant, Lifestyle Passive ^1^	Broadly Moderate Preventers ^1^	Compre-hensive High Engagers ^1^	Peripherally Engaged ^1^	Globally Low Engagers ^1^	*p*-Value ^2^	Cramer’s V ^3^
Gender						<0.001	0.241
Male	256 (44.4)	292 (58.6)	121 (29.2)	209 (55.2)	149 (66.5)		
Female	320 (55.6)	206 (41.4)	294 (70.8)	169 (44.8)	75 (33.5)		
Age Group						<0.001	0.144
18–34	93 (16.1)	135 (27.1)	92 (22.3)	128 (33.9)	47 (21.2)		
35–54	159 (27.6)	182 (36.5)	128 (30.9)	136 (36.0)	78 (34.8)		
55–99	324 (56.3)	181 (36.4)	194 (46.8)	114 (30.1)	98 (44.0)		
Education						<0.001	0.085
Middle school	129 (22.5)	116 (23.3)	62 (14.9)	79 (20.8)	55 (24.4)		
Vocational school	156 (27.1)	175 (35.0)	118 (28.5)	130 (34.4)	88 (39.3)		
High school	149 (25.9)	124 (24.9)	125 (30.2)	101 (26.6)	46 (20.8)		
University	141 (24.5)	84 (16.8)	110 (26.4)	69 (18.2)	35 (15.5)		
Language						<0.001	0.114
German	394 (68.5)	341 (68.5)	223 (53.8)	226 (59.8)	145 (64.9)		
Italian	131 (22.7)	82 (16.5)	136 (32.8)	85 (22.4)	46 (20.8)		
Other	50 (8.7)	75 (15.0)	55 (13.3)	67 (17.8)	32 (14.3)		
HLS-EU-Q16 category						<0.001	0.122
Inadequate	67 (14.0)	63 (17.5)	38 (10.7)	57 (19.8)	40 (24.0)		
Problematic	157 (32.8)	148 (40.9)	108 (30.5)	88 (30.9)	58 (34.4)		
Sufficient	254 (53.1)	150 (41.6)	208 (58.8)	141 (49.2)	70 (41.6)		
HLS-EU-Q16 score ^4^	12.2 (3.24)	11.4 (3.47)	12.6 (3.06)	11.7 (3.52)	11.0 (3.80)	<0.001	0.018
PAM-10						<0.001	0.127
Disengaged and overwhelmed	79 (13.8)	99 (19.9)	39 (9.5)	67 (17.7)	51 (22.6)		
Becoming aware but still struggling	235 (40.8)	219 (44.0)	168 (40.6)	158 (41.8)	107 (48.1)		
Taking action	139 (24.1)	122 (24.5)	80 (19.2)	99 (26.1)	50 (22.5)		
Maintaining behaviors and pushing further	123 (21.3)	58 (11.6)	127 (30.7)	54 (14.4)	15 (6.8)		
Chronic disease						0.008	0.085
No	350 (60.7)	351 (70.5)	270 (65.2)	254 (67.0)	132 (59.2)		
Yes	226 (39.3)	147 (29.5)	144 (34.8)	125 (33.0)	91 (40.8)		

^1^ Percentage (absolute count); due to population weighting and variable-specific missing data, the effective sample sizes used in statistical testing may differ from the unweighted totals indicated in the table header. ^2^ *p*-values were based on chi-square tests. ^3^ Effect sizes are presented as Cramér’s V; values are interpreted as follows: ≤0.10 = negligible, 0.10–0.19 = small, 0.20–0.29 = moderate, ≥0.30 = large effect. ^4^ Mean (SD), *p*-value of Kruskal–Wallis test; effect size estimated using eta squared; interpretation thresholds for η^2^: <0.01 = negligible, 0.01–0.06 = small, 0.06–0.14 = moderate, ≥0.14 = large.

**Table 3:** Population-based comparison of preventive health behavior between women and men (*n* = 2090).

Item	PHG-16 Score			*p*-Value ^2^	Cliff’s Delta ^3^
	Female Mean (SD) ^1^	Male Mean (SD) ^1^	Mean Difference		
1—Exercise regularly	3.23 (1.25)	3.24 (1.15)	0.01	0.482	−0.02
2—Follow a balanced diet	3.46 (1.11)	3.84 (1.02)	0.38	<0.001	−0.21
3—Take vitamins or dietary supplements	2.19 (1.19)	2.72 (1.28)	0.53	<0.001	−0.25
4—Visit the dentist regularly	3.34 (1.30)	3.77 (1.28)	0.43	<0.001	−0.21
5—Watch weight or try to lose weight	3.10 (1.19)	3.20 (1.18)	0.10	0.004	−0.07
6—Limit intake of high-fat or high-sugar foods	3.03 (1.06)	3.40 (1.01)	0.37	<0.001	−0.20
7—Gather health information before making decisions	2.85 (1.13)	3.37 (1.09)	0.52	<0.001	−0.24
8—Pay attention to physical symptoms or health changes	3.55 (1.12)	3.86 (1.07)	0.32	<0.001	−0.16
9—Take supplements to prevent illness	1.86 (1.14)	2.39 (1.31)	0.53	<0.001	−0.23
10—Get routine medical check-ups	2.82 (1.14)	3.20 (1.17)	0.38	<0.001	−0.19
11—Floss teeth regularly	2.46 (1.38)	2.92 (1.46)	0.45	<0.001	−0.18
12—Talk with others about health	2.79 (1.02)	3.29 (0.99)	0.49	<0.001	−0.26
13—Don’t smoke	4.20 (1.40)	4.36 (1.31)	0.17	0.002	−0.06
14—Brush teeth twice a day	4.61 (0.79)	4.83 (0.51)	0.21	<0.001	−0.12
15—Get vaccinated	3.40 (1.29)	3.54 (1.30)	0.14	0.012	−0.06
16—Get enough sleep	3.71 (0.94)	3.77 (0.95)	0.06	0.021	−0.05

^1^ Weighted means and SD. ^2^ Mann–Whitney U tests. ^3^ Effect size interpretation of Cliff’s delta: <0.147 = negligible effect, 0.147–0.33 = small effect, 0.33–0.474 = medium effect, ≥0.474 = large effect.

**Table 4:** Binary logistic regression predicting membership in Profile 5 ‘Globally Low Engagers’ versus Profile 2 ‘Broadly Moderate Preventers’ (*n* = 577; Profile 5 = 266; Profile 2 = 311).

Predictor	B	SE	Wald χ^2^	*p*	OR (Exp(B))	95% CI for OR
Intercept	0.508	0.684	0.550	0.458	1.662	[0.438, 6.300]
Gender (1 = Male)	0.416	0.251	2.748	0.097	1.516	[0.930, 2.469]
Age (continuous)	–0.017	0.006	8.303	0.004 **	0.983	[0.972, 0.994]
Education (ref = Middle school or lower)						
Vocational school	0.063	0.312	0.040	0.841	1.065	[0.577, 1.965]
High school	–0.296	0.337	0.774	0.379	0.744	[0.384, 1.443]
University	–0.016	0.406	0.002	0.968	0.984	[0.444, 2.181]
Language (ref = German)						
Italian	–0.453	0.325	1.942	0.163	0.636	[0.336, 1.202]
Other	–0.032	0.419	0.006	0.939	0.969	[0.426, 2.207]
Lives Alone (1 = Yes)	0.588	0.231	6.475	0.011 *	1.801	[1.144, 2.832]
Chronic Disease (1 = Yes)	0.582	0.201	8.391	0.004 **	1.789	[1.209, 2.647]
Health Literacy Score	–0.017	0.032	0.288	0.592	0.983	[0.923, 1.047]
Patient Activation Score	–0.010	0.008	1.522	0.217	0.990	[0.974, 1.006]
Mistrust Index	0.039	0.056	0.481	0.488	1.040	[0.932, 1.160]

Reference categories: Female (Gender), Middle school or lower (Education), German (Language), Not living alone (Lives Alone), No chronic disease (Chronic Disease). * *p* < 0.05, ** *p* < 0.01. Abbreviations: CI, Confidence Interval; SE, Standard Error; OR, Odds Ratio.

## Data Availability

Data are available from the corresponding author upon reasonable request.

## Abbreviations

The following abbreviations are used in this manuscript.

ASTAT	Provincial Institute of Statistics of South Tyrol
AvePP	Average Posterior Probabilities
BHC	Bolck-Croon-Hagenaars
BIC	Bayesian Information Criterion
BLRT	Bootstrap Likelihood Ratio Tests
CI	Confidence Interval
GHP-16	Good Health Practice 16 Items
HBC	Health Behavior Checklist
HBM	Health Belief Model
HLS-EU-Q16	Health Literacy Survey EU Questionnaire 16 Items
ISTAT	National Institute of Statistics of Italy
LMR	Lo-Mendell-Rubin
LPA	Latent Profile Analysis
PAM-10	Patient Activation Measure 10 Items
SD	Standard Deviation
TPB	Theory of Planned Behavior
VIF	Variance Inflation Factors
